# PHYSIOLOGICAL AGING AND ITS USE AS A MARKER OF INEQUALITIES IN HEALTH: CROSS-NATIONAL PERSPECTIVES

**DOI:** 10.1093/geroni/igae098.1219

**Published:** 2024-12-31

**Authors:** Mikaela Bloomberg

**Affiliations:** Chair

## Abstract

Given substantial heterogeneity in health status between older adults, there have been many efforts to develop metrics of biological and physiological ageing that explain variability in health outcomes better than chronological age alone. Physiological age based on clinical indicators may explain variation in health outcomes better than epigenetic or telomere-based biological clocks because clinical indicators capture downstream physiological changes more closely related to health outcomes. In this symposium, we examine physiological age as a holistic marker of ageing and how we can use different measures of physiological age to examine disparities in health across the UK, US, and Ireland. The first and second abstracts examine physiological ageing in different organ systems and associations with health outcomes in the US-based Health and Retirement Study (HRS) and the Irish Longitudinal Study on Ageing (TILDA). The third abstract explores gender and educational inequalities in physiological ageing in the English Longitudinal Study of Ageing (ELSA). The fourth examines racial differences in the returns to education for physiological ageing. Finally, the fifth abstract investigates associations of cumulative stress exposure and neighbourhood context with inflammation in HRS. We demonstrate physiological age as a powerful tool for revealing inequalities in health during ageing across national contexts.

